# Editorial: Molecular advances and new insights in aortic aneurysmal diseases

**DOI:** 10.3389/fcell.2026.1822834

**Published:** 2026-03-26

**Authors:** Yunwen Hu, Raffaele Serra, Cheng Wang, Jian Li, Zhaohua Cai

**Affiliations:** 1 Department of Cardiology, Shanghai Chest Hospital, Shanghai Jiao Tong University School of Medicine, Shanghai, China; 2 Interuniversity Center of Phlebolymphology (CIFL), International Research and Educational Program in Clinical and Experimental Biotechnology, University “Magna Graecia” of Catanzaro, Catanzaro, Italy; 3 Department of Medical and Surgical Sciences, University “Magna Graecia” of Catanzaro, Catanzaro, Italy; 4 Department of Rheumatology, Union Hospital, Tongji Medical College, Huazhong University of Science and Technology, Wuhan, China; 5 Institute for Biomedical Sciences, Georgia State University, Atlanta, GA, United States

**Keywords:** aortic aneurysm (AA), vascular smooth muscle cell (VSMC), phenotypic plasticity, perivascular adipose tissue (PVAT), animal model

Aortic aneurysms, traditionally defined by focal luminal dilation and progressive degeneration of the vascular wall, have long been regarded as localized vascular disorders. In this classical paradigm, aneurysmal disease is thought to result primarily from the convergence of focal vascular injury and hemodynamic insult, a conceptual framework that has historically shaped both experimental methodologies and clinical management (Wanhainen et al., 2019; Berger et al., 2025). Recognizing the need for a more comprehensive understanding of aneurysm pathobiology, the present Research Topic was launched to integrate emerging insights into vascular biology with clinical observations of aortic aneurysm progression. This collection comprises four contributions—two original research articles, one review, and one opinion piece—reflecting growing interest in elucidating both molecular and systemic mechanisms underlying aneurysmal disease. While it is indisputable that the vascular wall is constantly exposed to biomechanical forces, including pressure-induced tension, shear stress, and cyclic strain, accumulating clinical evidence challenges the sufficiency of a purely lesion-centric perspective. Notably, aortic aneurysms often continue to expand despite optimal blood pressure control and frequently manifest as multifocal or multi-territorial pathologies (Golledge and Singh, 2021), highlighting the need for a more integrated understanding of systemic mechanisms driving disease progression.

Recent advances in vascular biology have prompted a fundamental shift in our understanding of aneurysm pathogenesis. Aortic aneurysms are increasingly recognized not solely as a consequence of mechanical failure but as a systemic disruption of vascular homeostasis. In this evolving paradigm, the integrity of the vascular wall is maintained through dynamic processes involving cellular repair, adaptation, and phenotypic modulation of vascular smooth muscle cells (VSMCs). VSMCs, which are pivotal for preserving the structural and functional stability of the vessel wall, exhibit significant phenotypic plasticity in response to various environmental cues. Under physiological conditions, VSMCs predominantly adopt a contractile phenotype, characterized by high expression of contractile proteins and minimal extracellular matrix (ECM) production. A subset of VSMCs in the healthy aorta displays an adaptive phenotype, wherein contractile gene expression is downregulated while ECM production is upregulated (Li et al., 2020; Kan et al., 2021). This adaptive program is essential for maintaining vascular homeostasis under fluctuating biomechanical and biochemical conditions. Pathological states, however, can dysregulate this plasticity, driving VSMCs toward maladaptive phenotypes, including mesenchymal stem cell–like, fibroblast-like, and osteochondrocyte-like forms (Chen et al., 2020; Cai et al., 2024). These transitions initiate pathological remodeling cascades, encompassing ECM remodeling, elastic fiber fragmentation, and chronic low-grade inflammation, thereby creating a permissive microenvironment for further VSMC phenotypic changes and vascular degeneration.

VSMC phenotypic instability is not confined to isolated vascular segments but extends across broader arterial territories, contributing to the complex, multifocal nature of aneurysmal disease (https://doi.org/10.3389/fcell.2025.1592225). Within this globally destabilized vascular landscape, certain regions, most notably the infrarenal aorta—the segment of the abdominal aorta located distal to the renal arteries and proximal to the aortic bifurcation, display heightened susceptibility to aneurysm formation. While hemodynamic stress has traditionally been implicated in regional vulnerability, the relatively modest pressure gradients along the aorta suggest additional contributory factors. Emerging evidence indicates that regional heterogeneity in VSMC biology, including differences in embryological origin and interactions with local paravascular microenvironments, plays a key role in site-specific disease susceptibility. VSMCs derived from distinct embryological lineages, including somitic, lateral plate mesodermal, and neural crest origins, exhibit divergent transcriptional responses to stress, differential epigenetic landscapes, and distinct cell fate trajectories (Han et al., 2025). Local microenvironmental cues, particularly from perivascular adipose tissue, further modulate VSMC phenotypic stability and amplify regional heterogeneity in disease progression (Cai et al., 2025). Together, intrinsic VSMC heterogeneity and microenvironmental interactions drive the spatially heterogeneous patterns characteristic of aneurysmal disease.

At the molecular level, VSMC fate is orchestrated by intricate and highly integrated regulatory networks that coordinate mechanosensing, metabolic adaptation, stress signaling, and ECM dynamics. These networks are inherently heterogeneous, and perturbation in key regulatory genes, such as mutations in Transforming Growth Factor-β Receptor 1 (TGFBR1) or Transforming Growth Factor-β Receptor 2 (TGFBR2), can lead to widely divergent vascular phenotypes. The phenotypic consequences of these mutations depend on genetic context, mutation type, allelic dosage, and the presence of compensatory signaling pathways, contributing to the variability in aneurysm manifestation (https://doi.org/10.3389/fcell.2025.1580274). Emerging technologies, including machine learning and artificial intelligence, are proving invaluable for dissecting these complex networks (https://doi.org/10.3389/fcell.2025.1554972). By analyzing large-scale multi-omics datasets, these tools can uncover key regulatory nodes and molecular signatures that govern VSMC fate, offering novel insights into the underlying mechanisms and facilitating the development of targeted therapeutic strategies.

The growing body of evidence calls for a critical reassessment of experimental models used in aneurysmal research. Traditional models, which rely on chemical or mechanical injury to induce vascular decompensation, primarily replicate acute vascular disruption and thus fails to capture the chronic, progressive nature of human aneurysmal disease (Golledge et al., 2022). The molecular responses triggered by acute injury in animal models do not fully mirror the prolonged, evolving changes that characterize human aneurysm pathology, particularly with respect to VSMC phenotypic transitions and the temporal dynamics of signaling pathway activation (https://doi.org/10.3389/fcell.2025.1512938). In contrast, genetically defined models more faithfully reproduce the full spectrum of aneurysmal disease, from early molecular perturbations and diffuse arterial remodeling to multi-territorial ectasia and focal aneurysm formation. These models offer a biologically relevant platform for dissecting early pathogenic mechanisms and identifying stage-specific therapeutic targets (https://doi.org/10.3389/fcell.2025.1592225).

In conclusion, aortic aneurysm should be understood not as a localized structural failure, but as a manifestation of systemic disruption in the homeostatic mechanisms that govern vascular cell fate. VSMC phenotypic instability lies at the heart of this process, serving as both a driver and a barometer of disease progression. Elucidating the molecular programs that maintain or disrupt vascular cell identity will be central to advancing our understanding of aneurysm pathobiology and to developing more effective, mechanism-based interventions.
